# Comparative density of CCK- and PV-GABA cells within the cortex and hippocampus

**DOI:** 10.3389/fnana.2015.00124

**Published:** 2015-09-23

**Authors:** Paul D. Whissell, Janine D. Cajanding, Nicole Fogel, Jun Chul Kim

**Affiliations:** ^1^Department of Psychology, University of Toronto, TorontoON, Canada; ^2^Cell and Systems Biology, University of Toronto, TorontoON, Canada

**Keywords:** cholecystokinin, parvalbumin, interneuron, cortex, hippocampus, Dlx genes, intersectional genetics, balance

## Abstract

Cholecystokinin (CCK)- and parvalbumin (PV)-expressing neurons constitute the two major populations of perisomatic GABAergic neurons in the cortex and the hippocampus. As CCK- and PV-GABA neurons differ in an array of morphological, biochemical and electrophysiological features, it has been proposed that they form distinct inhibitory ensembles which differentially contribute to network oscillations and behavior. However, the relationship and balance between CCK- and PV-GABA neurons in the inhibitory networks of the brain is currently unclear as the distribution of these cells has never been compared on a large scale. Here, we systemically investigated the distribution of CCK- and PV-GABA cells across a wide number of discrete forebrain regions using an intersectional genetic approach. Our analysis revealed several novel trends in the distribution of these cells. While PV-GABA cells were more abundant overall, CCK-GABA cells outnumbered PV-GABA cells in several subregions of the hippocampus, medial prefrontal cortex and ventrolateral temporal cortex. Interestingly, CCK-GABA cells were relatively more abundant in secondary/association areas of the cortex (V2, S2, M2, and AudD/AudV) than they were in corresponding primary areas (V1, S1, M1, and Aud1). The reverse trend was observed for PV-GABA cells. Our findings suggest that the balance between CCK- and PV-GABA cells in a given cortical region is related to the type of processing that area performs; inhibitory networks in the secondary cortex tend to favor the inclusion of CCK-GABA cells more than networks in the primary cortex. The intersectional genetic labeling approach employed in the current study expands upon the ability to study molecularly defined subsets of GABAergic neurons. This technique can be applied to the investigation of neuropathologies which involve disruptions to the GABAergic system, including schizophrenia, stress, maternal immune activation and autism.

## Introduction

Inhibitory neurotransmission shapes neuronal activity and thereby regulates information processing (Freund, 2003). In the central nervous system, inhibition is predominantly mediated by interneurons signaling through the transmitter γ-aminobutyric acid (GABA; Kepecs and Fishell, 2014). Though interneurons constitute only a fraction of all neurons (∼15%) (Beaulieu, 1993), they are indispensable for multiple behavioral functions, including fear, anxiety, social interaction, olfaction, locomotion and memory (Cho et al., 2013; Donato et al., 2013; Courtin et al., 2014; Wolff et al., 2014). Imbalances in interneuron signaling have serious consequences for behavior, and have been implicated in the pathogenesis of neuropsychiatric disorders such as schizophrenia, Huntington’s disease, epilepsy and Alzheimer’s disease (Peng et al., 2013; Perez and Lodge, 2013; Aldrin-Kirk et al., 2014; Godavarthi et al., 2014; Kim et al., 2014a; Knoferle et al., 2014; Ma and McLaurin, 2014; Tong et al., 2014; Zamberletti et al., 2014). Collectively, interneurons represent a heterogeneous group of cells which differ in morphology, molecular composition, electrophysiological properties and distribution within the central nervous system (Kepecs and Fishell, 2014). Each of these interneuron subtypes may differentially contribute to behavioral function in health and disease (Lapray et al., 2012; Nguyen et al., 2014), and it remains a major challenge for neuroscience to characterize their specific properties.

Interneurons can be classified according to the targeting of their axonal projections to post-synaptic cells. Perisomatic interneurons, which account for nearly half of all hippocampal and cortical interneurons, form inhibitory synapses near the cell soma, proximal dendrites, and axon initial segments of their targets (Freund and Buzsaki, 1996). Perisomatic inhibition efficiently suppresses repetitive sodium-dependent action potentials (Miles et al., 1996) and entrains the network oscillations (Buzsaki and Wang, 2012) that accompany higher order cognitive operations (Wang, 2010). Perisomatic interneurons can be further subdivided into two separate groups based on the expression of the molecular markers cholecystokinin (CCK-GABA neurons) and parvalbumin (PV-GABA neurons). Notably, CCK- and PV-GABA neurons differ substantially in electrophysiological and biochemical features (Freund and Katona, 2007; Armstrong and Soltesz, 2012; Taniguchi, 2014). Briefly, CCK-GABA neurons exhibit moderate, accommodating firing patterns (Cauli et al., 1997) and express serotonin type 3 (5-HT3) receptors (Morales and Bloom, 1997) as well as cannabinoid type 1 (CB1) receptors (Katona et al., 1999). In contrast, PV-GABA neurons exhibit fast, non-accommodating firing rates (Cauli et al., 1997) and do not express either 5HT3 or CB1 receptors (Katona et al., 1999; Lee et al., 2010).

These contrasting features have stirred speculation that CCK- and PV-GABA neurons serve unique functions (Freund, 2003; Freund and Katona, 2007; Armstrong and Soltesz, 2012). PV-GABA neurons, which have been extensively studied, participate in gamma rhythm generation (Cardin et al., 2009; Sohal et al., 2009), neurogenesis (Song et al., 2013), sensory processing (Staiger et al., 1997, 2009; Moore and Wehr, 2013; Buetfering et al., 2014; Schneider et al., 2014; Siegle et al., 2014; Yekhlef et al., 2015), novelty recognition, associative learning and extinction of learned behavior (Letzkus et al., 2011; Donato et al., 2013; Bissonette et al., 2014; Courtin et al., 2014; Wolff et al., 2014). In comparison to PV-GABA neurons, CCK-GABA neurons have been less scrutinized and their functions are less clear. It has been argued that CCK-GABA neurons are ideally suited for the regulation of mood, anxiety and fear (Freund, 2003). Importantly, CCK-GABA neurons express 5HT3 and CB1 receptors, which are involved in mood regulation (Park and Williams, 2012; McLaughlin et al., 2014). Further, CCK-GABA neurons target post-synaptic regions that are enriched with receptors involved in anxiety behaviors, namely CCK type B and α2 subunit-containing GABA_A_ receptors (Nyiri et al., 2001; Raud et al., 2005; Koester et al., 2013). An important role for CCK-GABA neurons in anxiety and fear has been shown, though this function has been largely attributed to a modest population of neurons in the amygdala (Truitt et al., 2009; Brown et al., 2014; Schmidt et al., 2014; Bowers and Ressler, 2015). The functions of other populations of CCK-GABA neurons remain unclear.

Central to understanding the functions of CCK-GABA neurons is appreciating how these neurons are organized within the central nervous system. Whereas PV-GABA neuron distribution has been extensively characterized using an arsenal of techniques (Celio, 1986; del Rio et al., 1994; Tamamaki et al., 2003), CCK-GABA neuron distribution has received surprisingly little attention. Initially, CCK-GABA neuron distribution was inferred from antibody staining and autoradiography for CCK peptide and its messenger RNA (Innis et al., 1979; Savasta et al., 1988; Ingram et al., 1989). Unfortunately, such techniques are not selective for CCK-GABA neurons, as CCK is found in many glutamatergic neurons (Hokfelt et al., 2002). More recently, CCK-GABA neuron distribution in several regions of the hippocampus and cortex has been assessed through co-staining for CCK peptide and the GABA synthesizing enzyme glutamic acid decarboxylase (GAD) (Hendry et al., 1984; Demeulemeester et al., 1988; Kawaguchi and Kondo, 2002; Jinno and Kosaka, 2003a,b). While highly informative, these studies investigate CCK-GABA neuron distribution only in select regions of the hippocampus and cortex. It is currently unknown how the distribution of CCK-GABA neurons may vary in other regions.

The Cre recombinase-based genetic approach provides a powerful means to target a selective cell type using gene promoters with specific expression patterns, and may be of use in characterizing CCK-GABA neuron distribution. However, the specificity of this approach is limited by the availability of Cre transgenic lines. Further, cell populations defined by the Cre driver line alone are often heterogeneous. A recent study in CCK-Cre mice indicated that Cre activity is observed in both GABAergic and glutamatergic cells (Taniguchi et al., 2011), likely because CCK is expressed in both cell populations (Hokfelt et al., 2002). Therefore, the Cre recombinase-based genetic approach by itself is inadequate for the specific study of CCK-GABA neurons.

In the present study, we applied a dual recombinase-based genetic strategy to label CCK- and PV-GABA cells in the forebrain. The dual recombinase-based strategy labels a highly selective cell population which is defined by the overlapping expression pattern of two signature genes that drive the expression of Cre and Flpe recombinases. Specifically, Cre recombinase expression was driven by endogenous CCK or PV promoters whereas Flpe recombinase expression was driven by *Dlx5*/*Dlx6* intergenic regulatory sequences that are specific to forebrain GABA neurons, including those cells derived from both the medial and caudal ganglionic eminence (Miyoshi et al., 2010). Using a dual recombinase-responsive reporter allele, specific labeling of CCK- and PV-GABA cells in the forebrain was achieved by combining the Dlx5/6-Flpe allele with the CCK-Cre and PV-Cre allele, respectively. In mice with all three alleles, we then quantified the distribution of CCK- and PV-GABA cells in a wide volume of forebrain regions by unbiased automated cell counting methods.

## Materials and Methods

### Animals

Triple transgenic *CCK-Cre;Dlx5/6-Flpe;RC::FrePe* mice (termed CCK-*Frepe* mice) and *PV-Cre;Dlx5/6-Flpe;RC::FrePe* mice (termed PV-*Frepe* mice) were generated as follows: homozygous *RC::FrePe* mice (Bang et al., 2012; Robertson et al., 2013) were crossed with *Dlx5/Dlx6-FLPe* mice [Tg(mI56i-FLPe)39Fsh/J, JAX#010815] to generate double transgenic *Dlx5/6-Flpe;RC::FrePe* mice, which were then crossed with either homozygous CCK-ires-Cre mice [B6N.Cg-*Cck^tm1.1(cre)Zjh^*/J, JAX#019021] or PV-ires-Cre mice [B6;129P2-Pvalb*^tm1(cre)Arbr^*/J, JAX#008069]. Mice were group housed with *ad libitum* access to food and water in a temperature-controlled room on a 12 h light/dark cycle. Experimental procedures were in accordance with the guidelines of the Canadian Council on Animal Care (CCAC) and the local Animal Care Committee at the University of Toronto.

#### Immunohistochemistry

Triple transgenic mice 3–8 months old were selected for experiments. Mice were anesthetized with avertin and subsequently underwent transcardial perfusion with 0.1 M phosphate buffered saline (PBS; pH 7.4) followed by 4% paraformaldehyde (PFA) in PBS. Following perfusion, brains were extracted and placed in 4% PFA at 4°C for 24 h. Subsequently, brains were cryoprotected in a PBS solution containing 30% sucrose at 4°C for 48 h. Cryoprotected brains were then cut into 40 μM sections using a cryostat (CM1520; Leica) held at -20°C. From each brain, five sections were obtained from the intermediate frontal cortex (Bregma = 1.72 to 1.48 mm), intermediate parietal cortex (Bregma = -1.34 to -1.94 mm) and rostral occipital cortex (Bregma = -2.46 to -3.28 mm) (15 sections total; Paxinos and Franklin, 2012).

For fluorescent immunohistochemistry in cell counting experiments, free-floating tissue sections were rinsed with 0.1 M PBS and blocked with 5% normal donkey serum in 0.1% Triton-X-100 PBS (PBS-T) for 1 h at room temperature. Subsequently, sections were incubated with chicken polyclonal anti-green fluorescent protein (GFP; 1: 1000; ab13970; Abcam, Cambridge, MA, USA) and rabbit polyclonal anti-mCherry (1:1000; ab167453; Abcam) primary antibodies in PBS-T for 48 h at 4°C. Thereafter, sections were rinsed with PBS-T and incubated with Alexa 488-conjugated donkey anti-chicken (1:1000; 703545145; Jackson ImmunoResearch; West Grove, PA, USA) and Alexa 594-conjugated donkey anti-rabbit (1:1000; 715515152; Jackson ImmunoResearch) secondary antibodies in PBS-T for 2 hr at room temperature. Sections were then rinsed with PBS-T and mounted on Superfrost Plus slides (Fisher Scientific, Pittsburgh, PA, USA) and coverslipped with Aquamount (Polysciences Inc., Warrington, PA, USA).

In experiments examining the colocalization of CCK or PV with GFP, separate sections were incubated with chicken polyclonal anti-GFP antibody (1:1000; ab13970; Abcam) and either rabbit polyclonal anti-CCK-8 antibody in CCK-*Frepe* brain sections (1:1000; C2581; Sigma Aldrich; St. Louis, MO, USA) or mouse monoclonal anti-PV antibody in PV-*Frepe* brain sections (1:1000; ab11427; Abcam) in PBS-T for 48 h at 4°C. This was followed by incubation in PBS-T with Alexa 488-conjugated donkey anti-chicken antibody (1:1000; 703545145; Jackson ImmunoResearch) and either Alexa 405-conjugated donkey anti-rabbit antibody (1:1000; ab175651; Abcam) in CCK-*Frepe* brain sections or Alexa 405-conjugated donkey anti-mouse antibody (1:1000; ab175658; Abcam) in PV-*Frepe* brain sections for 2 h at room temperature.

In experiments examining the colocalization of GFP or mCherry with GAD67, CCK- and PV-*Frepe* sections were incubated with chicken polyclonal anti-GFP antibody (1:1000; ab13970; Abcam), goat polyclonal anti-mCherry antibody (1:1000; AB0040-20; Cedarlane, Burlington, ON, Canada) and mouse monoclonal anti-GAD67 antibody (1:1000; MAB5406; Billerica, MA, USA) in PBS-T for 48 h at 4°C. Sections were then incubated in PBS-T with Alexa 488-conjugated donkey anti-chicken antibody (1:1000; 703545145; Jackson ImmunoResearch), Alexa 594-conjugated donkey anti-goat antibody (1:1000; 705515147; Jackson ImmunoResearch), and Alexa 405-conjugated donkey anti-mouse antibody (1:1000; ab175658; Abcam) for 2 h at room temperature.

#### Image Acquisition and Cell Counting

For cell counting experiments with wide field fluorescent microscopy, images of brain sections were generated using an FSX100 fluorescent microscope (Olympus). Alexa 488 and 594 signals were captured using an U-MWIBA3 filter cube (Ex460-495, Em510-550, DM505) for Alexa 488 and an U-MWIG3 filter cube (Ex530-550, Em575IF, DM570) for Alexa 594. In acquired images, forebrain regions of interest (**Table 1**) were delineated manually according to area definitions established by Paxinos and Franklin (2012). Automated cell counting of GFP- and mCherry-labeled cells was performed using cellSens 1.7 software (Olympus), which determined the total number and numeric density of GFP- and mCherry-labeled cells in delineated brain regions. Cell density for each brain region was expressed in labeled cells/mm^2^. As 15 sections per animal were analyzed, multiple cell counts were available for every brain region. For each animal, we calculated a single value per brain region by averaging all values for that brain region across all sections.

**Table 1:** Distribution of CCK- and PV-GABA cells within the hippocampus and cortex.

			Density of GFP-labelled cells (cells/mm^2^)				% GFP-labelled cells/Total labelled cells				
			CCK-*Frepe*		PV-*Frepe*		CCK-*Frepe*		PV-*Frepe*		
			(*n* = 7)		(*n* = 4)		(*n* = 7)		(*n* = 4)		
Region	Subregion	Full name	Mean	SEM	Mean	SEM	Mean	SEM	Mean	SEM	*D* score
Hippocampus	dCA1	CA1, dorsal	61.6	7.5	35.5	4.8	31.7	3.0	16.4	3.2	15.3
	dCA3	CA3, dorsal	51.7	6.2	50.3	11.3	25.7	3.1	23.3	6.2	2.4
	dDG	Dentate gyrus, dorsal	20.5	2.8	19.6	1.3	16.1	2.2	12.8	2.0	3.3
	dSub	Subiculum, dorsal	42.1	3.4	108.1	10.1	22.7	2.6	37.0	4.8	-14.3
	vCA1	CA1, ventral	92.4	15.4	43.2	3.9	29.0	3.8	14.6	3.1	14.4
	vCA3	CA3, ventral	81.9	12.6	72.6	19.0	25.4	3.8	20.4	6.5	5.0
	vDG	Dentate gyrus, ventral	30.1	4.6	6.6	0.6	9.5	1.4	2.7	0.9	6.8
Frontal	Cg	Cingulate cortex	63.9	7.3	87.9	5.5	21.6	1.9	23.1	1.5	-1.5
	DP	Dorsal peducunlar region	73.3	11.8	41.3	7.4	24.7	3.2	10.4	0.8	14.2
	IL	Infralimbic cortex	75.4	9.7	34.9	2.6	21.4	2.5	7.5	0.6	13.9
	M1	Motor cortex, Primary	56.4	8.1	109.1	24.7	19.7	2.5	31.6	1.7	-11.8
	M2	Motor cortex, Secondary	70.2	7.1	100.2	13.2	24.1	2.9	30.1	1.3	-6.1
	PL	Prelimbic cortex	69.9	6.9	49.1	7.0	19.7	1.7	10.7	1.5	8.9
Retrosplenial	Rsd	Retrosplenial cortex, dysgranular	40.9	6.3	193.8	27.8	16.1	2.9	43.9	3.8	-27.8
	RsgA	Retrosplenial cortex, granular region A	42.4	7.4	95.3	17.0	21.4	4.7	27.6	4.3	-6.2
	RsgB	Retrosplenial cortex, granular region B	31.0	4.2	137.9	34.0	13.5	2.0	31.1	5.6	-17.6
	RsgC	Retrosplenial cortex, granular region C	33.7	6.6	205.4	43.3	14.1	3.1	44.5	5.2	-30.5
Parietal	PtaL	Parietal Association Area, Lateral	71.1	4.6	166.1	20.5	20.3	2.5	35.0	3.2	-14.7
	PtaM	Parietal Association Area, Medial	60.5	4.3	161.9	17.5	20.7	3.2	33.5	2.3	-12.8
	PtaP	Parietal Association Area, Posterior	49.6	8.3	155.3	18.2	15.1	2.7	36.8	4.1	-21.7
	S1	Somatosensory Area, Primary	39.9	6.4	148.7	19.1	13.3	2.4	39.3	6.6	-26.0
	S1bf	Somatosensory Area, Primary (barrel)	40.7	6.1	177.3	29.3	12.5	2.1	42.5	5.4	-30.1
	S1tr	Somatosensory Area, Primary (trunk)	63.5	8.9	175.2	26.3	17.6	2.9	40.3	5.1	-22.7
	S1ulp	Somatosensory Area, Primary (upper-lip)	33.8	6.7	146.1	11.6	11.7	2.4	39.4	3.6	-27.7
	S2	Somatosensory Area, Secondary	38.5	5.2	128.2	3.8	13.4	2.1	35.0	2.2	-21.6
Occipital	V1b	Visual cortex, Primary Basal	24.1	3.7	178.8	22.1	7.5	1.2	41.6	5.7	-34.0
	V1m	Visual cortex, Primary Medial	29.5	6.0	173.3	18.1	8.7	1.7	39.1	5.5	-30.4
	V2l	Visual cortex, Secondary Lateral	45.1	8.8	164.4	13.2	13.8	2.6	37.3	3.9	-23.5
	V2ml	Visual cortex, Secondary Mediolateral	55.2	9.6	136.1	12.0	15.1	2.7	30.9	4.4	-15.8
	V2mm	Visual cortex, Secondary Mediomedial	57.8	7.7	101.9	9.8	17.3	2.4	23.6	1.6	-6.3
Temporal	Aud1	Auditory cortex, Primary	28.3	4.0	147.6	10.0	9.8	1.6	36.7	2.6	-26.8
	AudD	Auditory cortex, Dorsal	44.5	6.4	139.2	9.1	14.1	2.4	36.1	3.7	-21.9
	AudV	Auditory cortex, Ventral	38.9	4.6	109.4	8.1	14.1	2.0	28.7	2.4	-14.7
	Ect	Ectorhinal cortex	34.6	6.2	21.6	2.0	17.8	2.9	13.9	1.0	4.0
	EntDI	Entorhinal cotex, Dorsointermediate	32.1	4.2	18.4	4.1	15.3	3.0	8.9	2.3	6.4
	EntDL	Entorhinal cortex, Dorsolateral	50.2	7.8	46.5	3.2	17.1	2.6	8.2	2.0	8.9
	PRh	Perirhinal cortex	43.7	5.2	43.0	3.2	19.6	2.5	15.2	1.5	4.4
	Tea	Temporal association area	53.3	8.2	58.1	7.4	18.1	2.8	15.0	1.4	3.1

*In each brain region, the density of CCK- and PV-GABA cells (units/mm^2^) and their relative contribution to the GABA cell population (%) is shown. Also included below is the ‘balance’ between CCK- and PV-GABA cells in a given brain region (*D*), expressed as the subtractive difference between CCK-GABA and PV-GABA cell percentage.*

For colocalization experiments, images were captured through a Quorum spinning disk confocal microscope (Zeiss) using a 20x objective lens and were subsequently analyzed with Volocity Software (Perkin Elmer). Alexa Fluor 405, 488, and 594 (secondary antibody signals) were excited with the 405, 491, and 561 nm laser, respectively.

In experiments examining the colocalization of CCK or PV with GFP, four sections per animal were counted from a 725 μm^2^ area in the CA1 subfield of the hippocampus. Cells were counted in the *stratum radiatum* in CCK-*Frepe* brain sections and the *stratum pyramidale* from PV-*Frepe* brain sections, as these areas show the highest density of CCK- and PV-GABA cells in the hippocampus, respectively. The specificity of our dual-recombinase approach was assessed by determining the percentage of GFP-labeled cells that were immunopositive for CCK (in CCK-*Frepe* mice) or PV (in PV-*Frepe* mice).

Similarly, in experiments examining the colocalization of GFP or mCherry with GAD67, sections were counted from CCK-*Frepe* (*n* = 2) and PV-*Frepe* mice (*n* = 2) in the *stratum radiatum.* We determined the percentage of fluorescently labeled cells (either GFP or mCherry) that were immunopositive for GAD67, and the percentage of GAD67-labeled cells also positive for either GFP or mCherry.

#### Statistical Analysis

All analysis was performed using GraphPad Prism 6.0 for Windows. Separate two-way analysis of variance (ANOVA) tests were performed for structures belonging to the hippocampus, frontal cortex, retrosplenial cortex, parietal cortex, occipital cortex and temporal cortex. In each two-way ANOVA, mouse genotype was used as a between-subjects factor (either CCK-*Frepe* or PV-*Frepe*) while brain region was used as a within-subjects factor. *Post-hoc* analysis was performed using Sidak’s multiple comparison test with the level of significance set at *p* = 0.05. When analyzing the percentage of GFP-labeled cells in each layer of the hippocampus, separate one-way ANOVA tests were performed for each subfield (CA1, CA3, and DG).

## Results

### Intersectional Genetic Labeling of CCK-GABA and PV-GABA Cells

**FIGURE 1:**
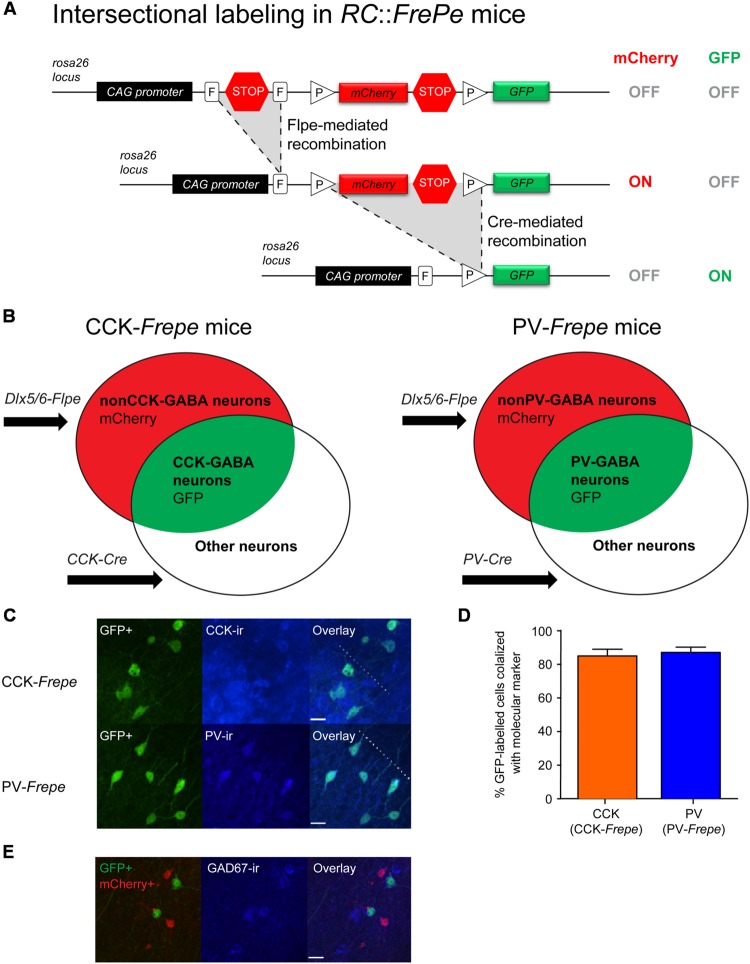
**Selective labeling of CCK- and PV-GABA cells using the dual recombinase–based intersectional genetic strategy. (A)** Top. A dual recombinase-responsive reporter allele, *RC::FrePe*, contains two transcriptional stop cassettes. The first stop cassette is flanked by vertically oriented FRT sites (rectangles, denoted with *F*) and the other by directly oriented loxP sites (triangles, denoted with *P*). The loxP-flanked stop cassette also contains mCherry-encoding sequences. Middle: Flpe-mediated stop cassette removal results in mCherry expression. The remaining loxP-flanked stop cassette prevents GFP expression. Bottom: Upon removal of both stop cassettes, requiring Flpe- and Cre-mediated excisions, expression of GFP is turned on and expression of mCherry is turned off. The *RC::FrePe* allele is knocked-in to the Gt(ROSA)26Sor (R26) locus with CAG (chicken β-actin and CMV enhancer) promoter elements. **(B)** Venn diagrams illustrating intersectional and subtractive cell populations labeled by the intersectional approach using the *RC::FrePe* allele. The Dlx5/6-Flpe allele is specific to GABAergic cells in the forebrain. In triple transgenic mice inheriting all three alleles (*CCK-Cre;Dlx5/6-Flpe;RC::FrePe* mice or *PV-Cre;Dlx5/6-Flpe;RC::FrePe* mice), cells expressing both Cre and Flpe alleles (i.e., intersectional population) represent CCK- or PV-GABA cells, and are labeled with GFP. In contrast, cells expressing only Flpe (i.e., subtractive population) represent nonCCK-GABA or nonPV-GABA neurons and are labeled with mCherry. **(C,D)** Specificity of labeling GABA cells using the intersectional genetic strategy. **(C)** Confocal images of the CA1 *stratum radiatum* in CCK-*Frepe* mice (top) and the CA1 *stratum pyramidale* in PV-*Frepe* mice (bottom). GFP-expressing cells are labeled in green whereas CCK+ cells (top) and PV+ cells (bottom) are shown in blue. The border between the *stratum pyramidale* and *stratum radiatum* is denoted by the dotted line. Scale bar = 20 μm. **(D)** Percentage of GFP-expressing cells in CCK- and PV-*Frepe* mice that are CCK+ (*orange*) or PV+ (*blue*), respectively. **(E)** Efficacy of GABA cell labeling in the intersectional genetic approach. Confocal image shows the CA1 *stratum radiatum* in PV-*Frepe* mice. GFP- and mCherry-expressing cells are shown in green and red, respectively, whereas GAD67+ cells are shown in blue. Scale bar = 20 μm.

To selectively label CCK- and PV-GABA cells for counting, we employed a dual recombinase-based intersectional genetic strategy (Jensen and Dymecki, 2014) (**Figure 1**). We first crossed the *Dlx5/6*-Flpe mouse line, which provides a selective genetic access to forebrain GABAergic neurons, with a dual recombinase-responsive reporter line, *RC::FrePe* (Engleka et al., 2012) (**Figure 1A**). Subsequently, double transgenic *Dlx5/6*-Flpe*;RC::FrePe* mice were crossed with either CCK-Cre or PV-Cre mouse lines, in which the expression of Cre recombinase is restricted to CCK and PV neurons, respectively. The resulting triple transgenic *CCK-Cre;Dlx5/6-Flpe;RC::FrePe* mice (CCK-*Frepe, n* = 7) and *PV-Cre;Dlx5/6-Flpe;RC::FrePe* mice (PV-*Frepe, n* = 4) were used in cell counting experiments (**Figure 1B**). In CCK- and PV-*Frepe* mice, cells expressing Flpe and Cre recombinase (i.e., CCK- or PV-GABA cells) are labeled with reporter enhanced green fluorescent protein (GFP) (**Figure 1B**). In contrast, cells expressing only Flpe recombinase (i.e., nonCCK-GABA and nonPV-GABA cells) are labeled with the reporter protein mCherry. Cells expressing Cre recombinase alone, or neither recombinase, are unlabeled.

Prior to cell counting, we examined the specificity of labeling in the intersectional genetic approach. In CCK- and PV-*Frepe* mice, GFP reporter expression was compared with CCK and PV immunofluorescence, respectively (**Figures 1C,D**). In sections from CCK-*Frepe* mice (*n* = 4), 85.0% of GFP-expressing cells were positive for CCK immunoreactivity. In sections from PV-*Frepe* mice (*n* = 4), 87.1% of GFP-expressing cells were positive for PV immunoreactivity. These data indicate that the large majority of CCK- and PV-GABA cells are labeled with GFP in CCK- and PV-*Frepe* mice, and verify a high specificity of labeling using the intersectional approach.

Furthermore, we examined the efficacy of the intersectional genetic approach for labeling GABA cells. Previous studies have shown that the *Dlx5/6*-Flpe line can be used to label the majority of GABA cells derived from the medial, caudal and lateral ganglionic eminences (Miyoshi et al., 2010). One study reported that as many as ∼90% of GABA cells in the neocortex may be labeled by combining the *Dlx5/6*-Flpe line with a rosa26 knock-in Flpe reporter line (Imayoshi et al., 2012). To determine the efficacy of GABA cell labeling, we quantified the percentage of fluorescently labeled cells (either with GFP or mCherry) that were immunopositive for the enzyme glutamic acid decarboxylase 67 (GAD67), which is a marker of GABA neurons (**Figure 1E**). In a sample of CCK-*Frepe* (*n* = 2) and PV-*Frepe* brains (*n* = 2), we observed that 71.5% of fluorescently labeled cells in the hippocampus were also positive for GAD67. Additionally, we observed that 70.0% of GAD67-positive cells were also positive either GFP or mCherry. These data verify that the majority of GABA cells are labeled using the intersectional approach.

### Hippocampus

To further validate the effectiveness of the intersectional genetic approach, we quantified GFP-labeled cells in the hippocampus of CCK- and PV-*Frepe* mice. We reasoned that this structure would provide an ideal reference point as it is populated by many GABAergic neurons, including CCK- and PV-GABA neurons, of well-characterized distribution (Kosaka et al., 1985, 1987; Nomura et al., 1997; Jinno and Kosaka, 2006). Additionally, the hippocampus is a convenient target for histological examination as its circuitry and anatomy have been extensively studied (Freund and Buzsaki, 1996; Strange et al., 2014). Thus, we tabulated the percentage of GFP-labeled cells within the hippocampus of CCK- and PV-*Frepe* mice, and compared these values to those established for CCK- and PV-GABA cells using other labeling techniques.

**FIGURE 2:**
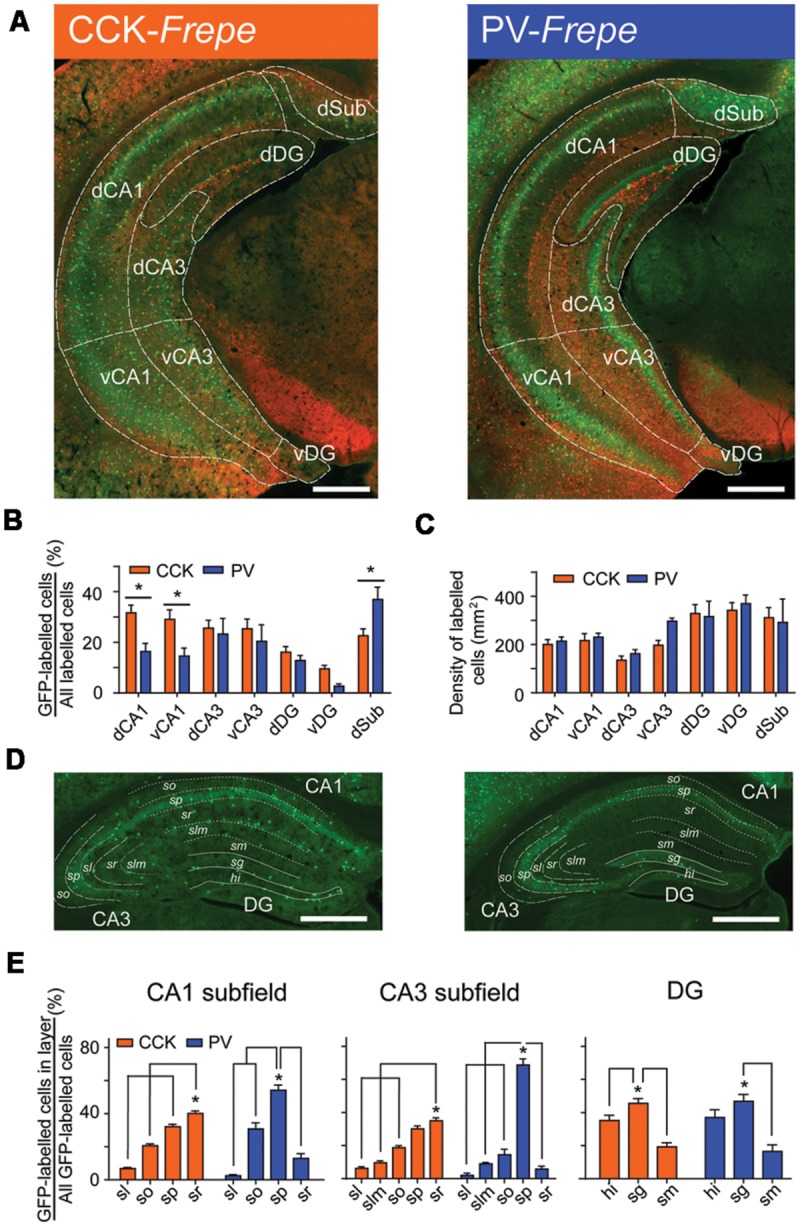
**Distribution of CCK- and PV-GABA cells within the hippocampus. (A)** Hippocampal sections from CCK-*Frepe* (left) and PV-*Frepe* mice (right). GFP-labeled cells represent CCK- and PV-GABA cells, respectively. **(B)** Percentage contribution of CCK-GABA cells (*orange*) and PV-GABA cells (*blue*) to the GABA neuron population in each hippocampal subregion. CCK-GABA cells were comparatively more abundant in the dCA1 and vCA1 regions whereas PV-GABA cells were more abundant in the dSub region. **(C)** The total density of GABA cells, defined as the sum of GFP- and mCherry-labeled cell density, did not differ between CCK- and PV-*Frepe* mice. **(D,E)** Distribution of CCK- and PV-GABA cells by hippocampal layer. CCK-GABA cells were most common in the sr layer but were also found in the sp and so layers. PV-GABA cells were more concentrated in the sp layer, but were also found in the so layer. *Abbreviations by subregion: dCA1*, dorsal CA1, *dCA3*, dorsal CA3, *dDG*, dorsal dentate gyrus, *dSub*, dorsal subiculum, *vCA1*, ventral CA1, *vCA3*, ventral CA3 and *vDG*, ventral dentate gyrus. *Abbreviations by layer: hi*, hilus, *sg*, stratum granulosum, *sl*, stratum lucidum, *slm*, stratum lacunosum-moleculare, *sm*, stratum moleculare, *so*, stratum oriens, *sp*, stratum pyramidale and *sr*, stratum radiatum. Significance at the *p* < 0.05 level is denoted with an asterisk. Scale bar = 500 μM.

In CCK- and PV-*Frepe* mice, cells labeled with GFP or mCherry were quantified using unbiased automated cell counting. The percentage of CCK- and PV-GABA cells was defined as the percentage of fluorescently labeled cells that were GFP-labeled cells (i.e., GFP cells/[GFP cells + mCherry cells]). In our analysis of the hippocampus, we counted labeled cells in the *Cornu Ammonis* subfields (CA1, CA3), dentate gyrus (DG) and subiculum (Sub). As the dorsal and ventral hippocampus are often considered anatomically and functionally distinct (Nomura et al., 1997; Strange et al., 2014), dorsal and ventral subregions were defined separately by using the rhinal fissure as a landmark. Subregions above the rhinal fissure were considered to belong to the dorsal hippocampus whereas subregions below were considered to belong to the ventral hippocampus. In total, eight hippocampal subregions were counted (dCA1, dCA3, dDG, dSub, vCA1, vCA3, vDG, and vSub) (**Table 1**, **Figure 2**). As our sampling method focused on the intermediate sections of the mouse brain, the caudal vSub was often absent. Accordingly, this region was excluded from analysis.

The density and percentage of GFP-labeled cells within hippocampal subregions of CCK- and PV-*Frepe* mice is shown in **Table 1**. Two-way ANOVA detected a significant interaction of genotype × brain region on the percentage of GFP-labeled cells [*F*(6,62) = 3.72, *p* < 0.01]. In both the dCA1 and vCA1 subfields, the percentage of CCK-GABA cells was greater than PV-GABA cells (*p* < 0.05, **Figures 2A,B**). The relative abundance of CCK-GABA cells within the ventral hippocampus of CCK-*Frepe* mice is consistent with past reports, which show a relatively high density of CCK-GABA cells in this region (Jinno and Kosaka, 2003b, 2006; Meyer, 2014). Further, we observed that PV-GABA cells were more numerous in the dSub (*p* < 0.05, **Figures 2A,B**), as shown previously (Pitkanen and Amaral, 1993; Boccara et al., 2014). In the remaining subregions of the hippocampus (dCA3, vCA3, dDG, and vDG), the percentages of CCK- and PV-GABA cells did not differ (all *p*s > 0.05, **Figures 2A,B**). The observed differences in the percentage of CCK- and PV-GABA cells across hippocampal subregions were not due to differences in total GABA cell count within the hippocampus, as the total density of labeled cells (GFP + mCherry) did not differ between CCK- and PV-*Frepe* mice by subregion (*p* > 0.05, **Figure 2C**). Additionally, these counts were not due to an experimenter’s bias in the delineation of hippocampal subregions. When hippocampal cell counts were re-analyzed by an experimenter blind to condition, total GABA cell count did not differ in any subregion between CCK- and PV-*Frepe* mice (all *p*s > 0.05, data not shown).

Collectively, these data show that the distribution of GFP-labeled cells in CCK- and PV-*Frepe* mice is consistent with the known distribution of CCK- and PV-GABA cells within the subregions of the hippocampus (Kosaka et al., 1985, 1987; Nomura et al., 1997; Jinno and Kosaka, 2006). However, the distribution of GABAergic cells also differs by hippocampal cell layer as well as by subregion (Kosaka et al., 1985; Schiffmann and Vanderhaeghen, 1991; Freund and Buzsaki, 1996; Botcher et al., 2014). To examine the layer-specific patterns of distribution of CCK- and PV-GABA cells, we counted GFP-labeled cells within the layers of the hippocampus of CCK- and PV-*Frepe* mice. In a sample of dorsal hippocampal sections, GFP-labeled cells were quantified in the *stratum oriens* (CA1so, CA3so), *stratum pyramidale* (CA1sp, CA3sp), *stratum lacunosum-moleculare* (CA1slm, CA3slm), *stratum lucidum* (CA3sl), *stratum moleculare* (DGsm), *stratum granulosum* (DGsg) and *hilar region* (DGhi) (**Figures 2D,E**).

One-way ANOVA detected a significant effect of hippocampal layer on the percentage of GFP-labeled cells in the CA1 [CCK-*Frepe*: *F*(3,24) = 145.4, *p* < 0.0001; PV-*Frepe*: *F*(3,12) = 64.71, *p* < 0.0001], CA3 [CCK-*Frepe*: *F*(4,30) = 77.29, *p* < 0.0001; PV-*Frepe*: *F*(4,15) = 125.7, *p* < 0.0001] and DG subfields [CCK-*Frepe*: *F*(2,18) = 21.35, *p* < 0.0001; PV-*Frepe*: *F*(2,9) = 12.97, *p* < 0.01]. In the CA1 subfield, CCK-GABA cells were commonly found in the CA1so, CA1sp and CA1sr layers but not in the CA1slm layer (all *p*s < 0.05). The content of CCK-GABA cells was greatest in the CA1sr layer but was also high in the CA1sp layer, as shown previously (Jinno and Kosaka, 2006) (**Figures 2D,E**). A similar distribution of CCK-GABA cells was seen in the CA3 subfield, with GFP-labeled cells being more numerous in the CA3sr layer (all *p*s < 0.05, **Figures 2D,E**). In contrast, PV-GABA cells were highly concentrated in the sp layer of CA1 and CA3 (all *p*s < 0.05, **Figures 2D,E**), as shown by others (Jinno and Kosaka, 2002). In the DG, both CCK- and PV-GABA cells were numerous in the DGsg layer and infrequent in the DGml, as demonstrated previously (Jinno and Kosaka, 2002, 2003a,b). Overall, these data demonstrate that the pattern of GFP-labeling in the *Frepe* model is highly consistent with that shown by antibody labeling studies.

### Frontal Cortex

Next, GFP-labeled cells were quantified in the frontal cortex of CCK- and PV-*Frepe* mice. In this region, CCK is considered one of the dominant neuropeptides (Hornung et al., 1992) and many cells are positive for CCK messenger RNA (Savasta et al., 1988; Oeth and Lewis, 1993). While these findings might imply a large population of CCK-GABA neurons is present, early studies using antibody labeling suggested that such cells represented only a small proportion of GABAergic neurons (1–5%; Kubota et al., 1994; Kubota and Kawaguchi, 1997). In contrast, more recent research using single-cell reverse transcription-polymerase chain reaction (scRT-PCR) techniques suggests that many interneurons in the cortex express CCK mRNA (∼30–40%; Gallopin et al., 2006), though it is debated whether transcript expression reflects CCK-GABA neuron identity (Tricoire et al., 2011). These conflicting results have generated some controversy regarding the abundance and importance of CCK-GABA neurons in the frontal cortex. We reasoned that the intersectional approach, which labels a subpopulation of GABA neurons according to their genetic history for CCK expression, would provide insight into this situation.

**FIGURE 3:**
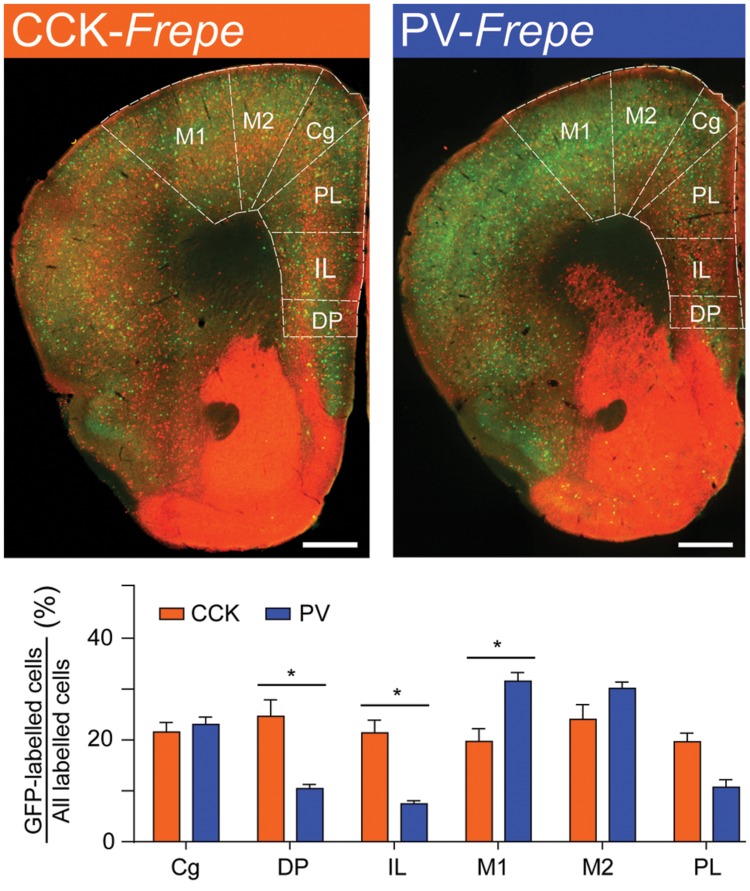
**CCK-GABA cells are most numerous within the medial prefrontal cortex. (Top)** Sections of the prefrontal cortex in CCK- and PV-*Frepe* mice. **(Bottom)** Percentage contribution of CCK- and PV-GABA cells to the total GABA neuron population by subregion of the frontal cortex. In the DP and IL regions, CCK-GABA cells were more abundant than PV-GABA cells. CCK-GABA cells also tended to be more abundant in the PL region. PV-GABA cells were more abundant in M1 region and tended to be more abundant in the M2 region. *Abbreviations: Cg*, cingulate cortex, *DP*, dorsal peduncular region, *IL*, infralimbic cortex, *M1*, primary motor cortex, *M2*, secondary motor cortex, *PL*, prelimbic cortex. Significance at the *p* < 0.05 level is denoted with an asterisk. Scale bar = 500 μM.

Within the frontal cortex, GFP-labeled cells were counted in the cingulate cortex (Cg), dorsal peduncular region (DP), infralimbic (IL) cortex, primary motor cortex (M1), secondary motor cortex (M2) and prelimbic (PL) cortex (**Figure 3**, **Table 1**). These particular subregions of the frontal cortex are relatively easy to distinguish, contain elaborate networks of CCK- and PV-GABA neurons, and are implicated in the pathogenesis of neurodegenerative and neuropsychiatric diseases (Bachus et al., 1997; Penschuck et al., 2006; Abdul-Monim et al., 2007; Braun et al., 2007; Falco et al., 2014; Uchida et al., 2014; Wischhof et al., 2015). Two-way ANOVA detected a significant interaction of genotype × brain region on the percentage of GFP-labeled cells [*F*(5,48) = 11.27, *p* < 0.001]. Within the IL and DP regions of the frontal cortex, CCK-GABA cells were more numerous than PV-GABA cells (*p*s < 0.05; **Figure 3**). In the PL, CCK-GABA cells also tended to be more numerous, though the difference was not significant. In contrast to the relative abundance of CCK-GABA cells in medial prefrontal cortex subregions (IL/PL/Cg), PV-GABA cells were significantly more abundant in the M1 subregion (*p*s < 0.05, **Figure 3**) and tended to be more abundant in M2 subregion. Collectively, PV-GABA cells constituted ∼40% of all GABAergic cells in M1 and M2, as shown previously (Conde et al., 1994; Tamamaki et al., 2003; Uchida et al., 2014). There were no differences in the percentages of CCK- and PV-GABA cells in the Cg (*p* > 0.05). In summary, these data suggest that CCK-GABA cells comprise a larger proportion of the GABAergic population in the medial prefrontal cortex than in the adjacent motor cortex.

### Retrosplenial Cortex

The retrosplenial cortex plays an important role in visuospatial processing, memory and extinction (Vann et al., 2009; Czajkowski et al., 2014; Kwapis et al., 2014; Nelson et al., 2015; Todd et al., 2015) but has received surprisingly little anatomical investigation, with only limited information suggesting abundant PV-GABA neurons (del Rio et al., 1994). As the circuitry of the retrosplenial cortex is still being unraveled (Vann et al., 2009), the contribution of other GABAergic neurons, including CCK-GABA neurons, needs to be characterized.

GFP-labeled cells were counted in the subdivisions of the retrosplenial cortex, including the dysgranular (Rsd) and granular regions (RsgA, RsgB and RsgC) (**Table 1**). Two-way ANOVA detected a significant interaction of genotype × brain region on the percentage of GFP-labeled cells [*F*(3,36) = 3.77, *p* < 0.05]. PV-GABA cells were significantly more numerous than CCK-GABA cells in the Rsd, RsgB, and RsgC subdivisions (*p* < 0.05). Additionally, PV-GABA cells tended to be more numerous in the RsgA subdivision but the difference was not significant. These data strongly suggest that PV-GABA neurons are by far the dominant perisomatic interneurons in the retrosplenial cortex.

### Parietal Cortex

The parietal cortex comprises a large portion of the mouse brain and contains dense networks of perisomatic interneurons (Hendry et al., 1983; Houser et al., 1983). PV-GABA neurons are especially numerous in the parietal cortex (Celio, 1986; Ren et al., 1992; del Rio et al., 1994; Powell et al., 2003; Xu et al., 2010; Perrenoud et al., 2013), where they regulate many functions, including the processing of sensory information from the thalamus regarding tactile perception (Staiger et al., 1997, 2009; Siegle et al., 2014). As antibody labeling studies have established that PV-GABA neurons are more abundant than most other GABA neurons in the parietal cortex, we expected PV-GABA cells to be highly numerous in the *Frepe* model.

**FIGURE 4:**
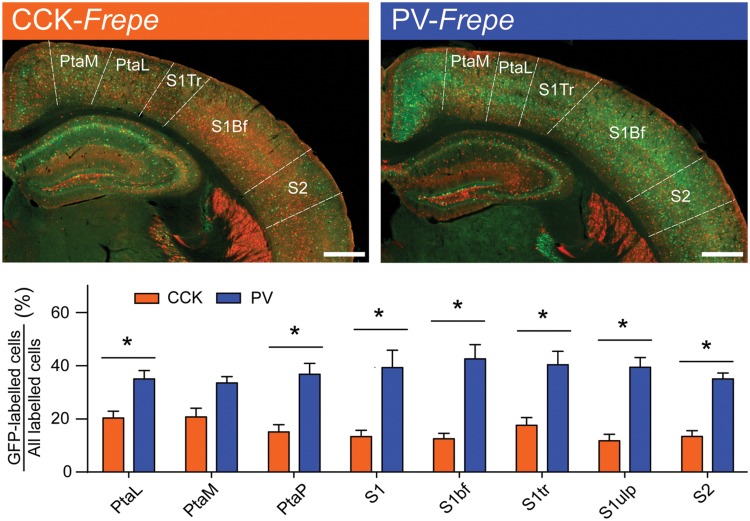
**Abundance of PV-GABA cells in the parietal cortex. (Top)** Sections of the intermediate parietal cortex from CCK- and PV-*Frepe* mice. **(Bottom)** Relative contribution of CCK- and PV-GABA cells to the total GABA neuron population by subregion of the parietal cortex. PV-GABA cells were comparatively more numerous in every subregion but the PtaM. *Abbreviations: PtaL*, lateral parietal association area, *PtaM*, medial parietal association area, *PtaP*, posterior parietal association area, *S1*, primary somatosensory area, *S1bf*, somatosensory area (barrel), *S1tr*, somatosensory area (trunk), *S1ulp*, somatosensory area (upper lip region), *S2*, secondary somatosensory area. Significance at the *p* < 0.05 level is denoted with an asterisk. Scale bar = 500 μM.

Due to the substantial volume of the parietal cortex, we elected to sample intermediate subregions only. Specifically, GFP-labeled cells were counted in several subdivisions of the primary somatosensory cortex (S1bf, S1tr, S1ulp, S1), parietal association cortex (PtaL, PtaM, and PtaP) and in the secondary somatosensory cortex (S2) (**Table 1**, **Figure 4**). Two-way ANOVA detected a main effect of genotype on the percentage of GFP-labeled cells in the parietal cortex [*F*(1,71) = 179.3, *p* < 0.0001], but not an interaction of genotype × brain region. In all regions of the parietal cortex but the PtaM, PV-GABA cells were significantly more numerous than CCK-GABA cells (all *p*s < 0.05, **Figure 4**). These data support the notion that PV-GABA cells are the most abundant perisomatic interneurons in the parietal cortex (Lee et al., 2010).

### Occipital Cortex

**FIGURE 5:**
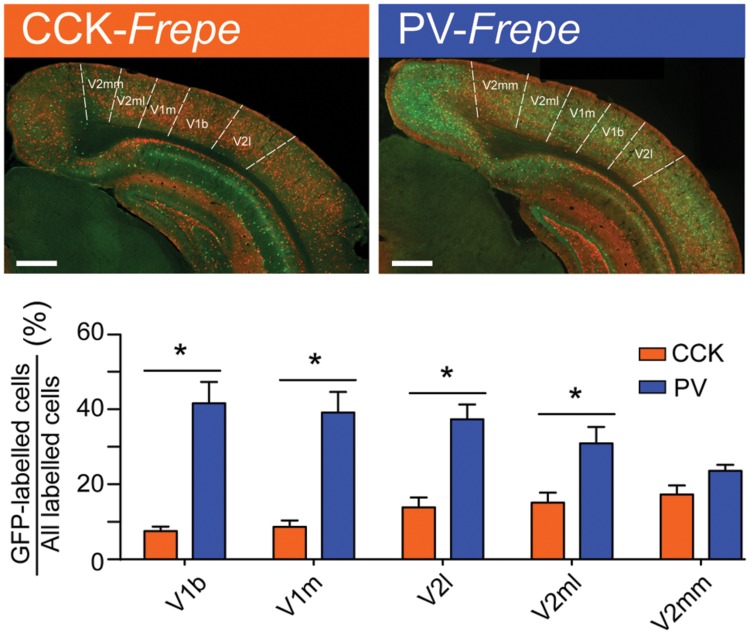
**Abundance of PV-GABA cells in the occipital cortex. (Top)** Sections of the occipital cortex from CCK- and PV-*Frepe* mice. **(Bottom)** Percentage contribution of CCK- and PV-GABA cells to the total GABA cell population by subregion of the occipital cortex. PV-GABA cells are comparatively more numerous in every subregion but the V2mm, where they tended to be more numerous. *Abbreviations: V1b*, primary visual cortex, basal region; *V1m*, primary visual cortex, medial region; *V2l*, secondary visual cortex, lateral region; *V2ml*, secondary visual cortex, mediolateral region; *V2mm*, secondary visual cortex, mediomedial region. Significance at the *p* < 0.05 level is denoted with an asterisk. Scale bar = 500 μM.

Similar to the parietal cortex, the occipital cortex is thought to include large quantities of PV-GABA neurons (∼40%) and low quantities of other GABAergic neurons (Demeulemeester et al., 1988; Hornung et al., 1992; Beaulieu, 1993; del Rio et al., 1994; Gonchar et al., 2007; Xu et al., 2010). However, most prior studies of the occipital cortex have focused on the primary visual cortex (V1) (Hornung et al., 1992; Beaulieu, 1993; del Rio et al., 1994) and have not extensively studied GABAergic cell content in other subregions, such as the secondary visual cortex (V2). In these areas, the relative distribution of GABAergic neurons is less clear. To determine whether the distribution of CCK- and PV-GABA cells varied across the occipital cortex, GFP-labeled cells were measured in the main subdivisions of the primary visual cortex (V1b, V1m) and secondary visual cortex (V2mm, V2ml, and V2l) (**Figure 5**).

Two-way ANOVA detected a significant interaction of genotype × brain region on the percentage of GFP-labeled cells [*F*(4,45) = 6.51, *p* < 0.01]. In all subdivisions of the occipital cortex but V2mm, PV-GABA cells were more numerous than CCK-GABA cells (all *p*s < 0.05, **Figure 5**). In the V2mm subregion, PV-GABA cells tended to be more abundant, but the difference was not significant according to *post hoc* analysis (*p* > 0.05). Interestingly, the observation that PV-GABA cells are less numerous in the V2mm area than in the surrounding visual cortices has also been made in a previous antibody labeling study for PV (del Rio et al., 1994). Overall, there was a prominent trend observed wherein CCK-GABA cells tended to be more numerous in secondary visual cortex regions (V2mm, V2ml, V2l) than in the primary visual cortex regions (V1b, V1m).

### Temporal Cortex and Association Areas

The temporal cortex is critical for auditory processing, multimodal associations and memory formation (Squire et al., 2004; Schreiner and Winer, 2007). This region contains large networks of GABAergic basket cells, many of which are thought to be PV-GABA neurons (Blazquez-Llorca et al., 2010). PV-GABA neuron networks of the temporal cortex have been extensively described (Celio, 1986; Pitkanen and Amaral, 1993; McMullen et al., 1994) and regulate many functions, including the processing of auditory information and spatial mapping (Moore and Wehr, 2013; Buetfering et al., 2014; Schneider et al., 2014; Yekhlef et al., 2015). Comparatively, there is little anatomical or functional understanding of CCK-GABA neuron networks within the temporal cortex. While antibody labeling studies suggest that many CCK-expressing neurons are indeed present, particularly within the ventrolateral temporal region (Innis et al., 1979; Miceli et al., 1987; Ingram et al., 1989), it is unknown whether these cells are CCK-GABA neurons specifically.

**FIGURE 6:**
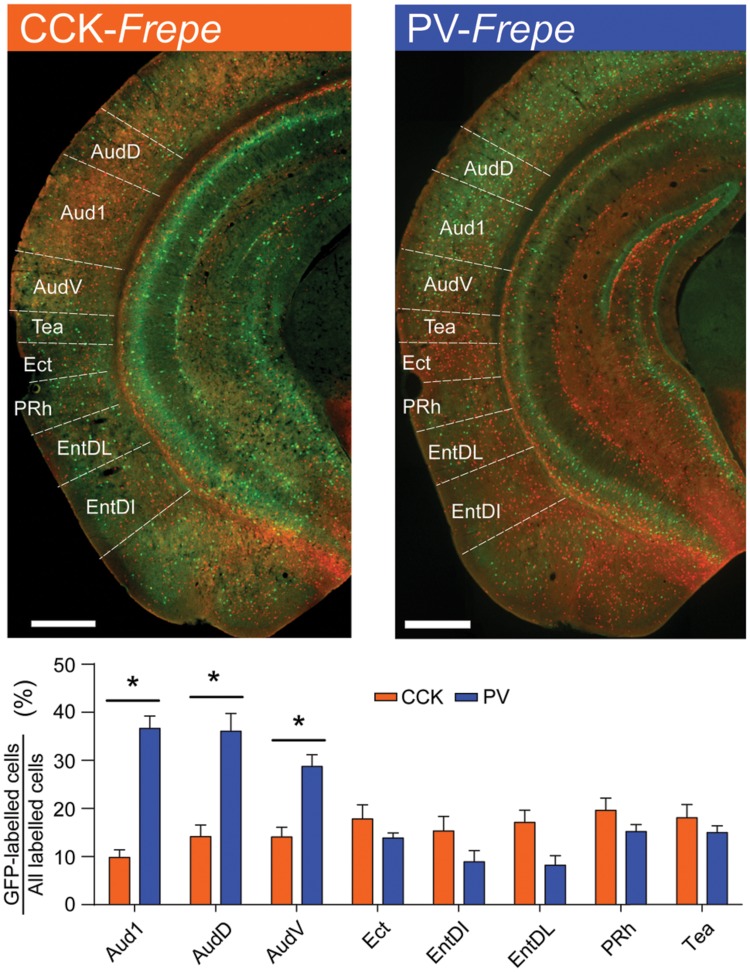
**Contrasting distribution of CCK- and PV-GABA cells in the temporal cortex. (Top)** Sections of the temporal cortex from CCK- and PV-*Frepe* mice. **(Bottom)** Percentage contribution of CCK- and PV-GABA cells to the total GABA cell population by subregion of the temporal cortex. PV-GABA cells are significantly more numerous in auditory cortex regions. Ventral to the rhinal fissure, PV-GABA cells become less common and CCK-GABA cells become more numerous. *Abbreviations: V1b*, primary visual cortex, basal region; *V1m*, primary visual cortex, medial region; *V2l*, secondary visual cortex, lateral region; *V2ml*, secondary visual cortex, mediolateral region; *V2mm*, secondary visual cortex, mediomedial region. Significance at the *p* < 0.05 level is denoted with an asterisk. Scale bar = 500 μM.

GFP-labeled cells in CCK- and PV-*Frepe* mice were enumerated in the intermediate regions of the temporal cortex. In our sampling method, we included subdivisions of the auditory cortex (Aud1, AudD, AudV) as well as the temporal association cortex (Tea), ectorhinal cortex (Ect), perirhinal cortex (Prh) and entorhinal cortex (EntDI and EntDL) (**Table 1**, **Figure 6**). Two-way ANOVA detected a genotype × brain region interaction on the percentage of GFP-labeled cells [*F*(7,71) = 14.45, *p* < 0.0001]. In all subdivisions of the auditory cortex, PV-GABA cells were highly abundant and more numerous than CCK-GABA cells (all *p*s < 0.05, **Figure 6**). PV-GABA cell density was much lower outside of the auditory cortex, as observed in other species (McMullen et al., 1994). Interestingly, the transition from the ventral auditory cortex (AudV) into the area of the rhinal fissure (Tea, Ect, and PRh) coincided with an abrupt reduction in PV-GABA cell percentage. Surprisingly, the reverse trend in distribution was observed for CCK-GABA cells, which tended to be more numerous in the Tea, Ect, and PRh areas than in auditory cortex. As a result of these contrasting trends, the percentage count of CCK- and PV-GABA cells was comparable in temporal association areas. In fact, CCK-GABA cell percentage even tended to be higher than PV-GABA cell percentage in the examined subdivisions such as the entorhinal cortex (EntDI, EntDL), though the difference was not significant.

### Comparative Distribution of CCK- and PV-GABA Cells

**FIGURE 7:**
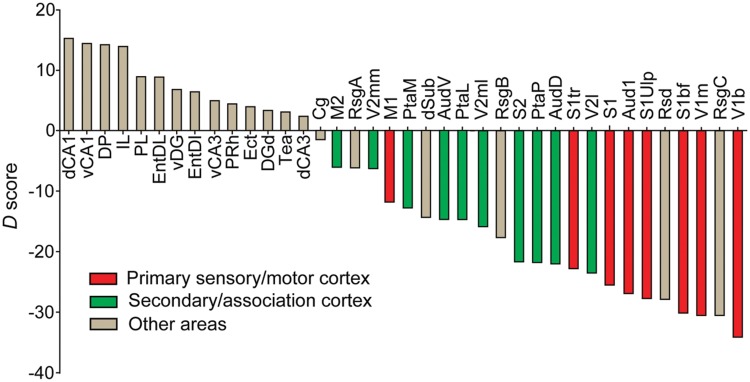
**Perisomatic interneuron balance and its relationship to cortical function.** Cell balance was estimated using the delta score (*D*), which was the subtractive difference between CCK-GABA and PV-GABA cell percentage. Brain regions with *D* > 0 had more CCK-GABA cells, whereas regions with *D* < 0 had more PV-GABA cells. Moving right along *x*-axis of the graph, a decreasing *D* reflects a relatively increasing PV-GABA cell content. Regions of the primary sensory cortex or primary motor cortex (*red*) had negative *D*, indicating relatively higher PV-GABA cell content. Interestingly, secondary regions (*green*) had less negative *D* than their primary counterparts, indicating relatively lower PV-GABA cell content. For comparison, other non-motor and non-sensory cortical subregions are included (*gray*).

As CCK- and PV-GABA neurons are thought to regulate pyramidal cell activity by distinct mechanisms (Freund, 2003; Freund and Katona, 2007), they may be specialized for certain cortical network activities. Within the cortex, information processing is known to differ substantially between the primary and secondary regions. Primary cortical regions (such as Aud1, V1, S1, and M1) are typically situated early in hierarchal networks (Van Essen et al., 1992; Kaas et al., 1999; Kim et al., 2014b), and must rapidly transmit information to other brain regions for higher-order processing. In comparison, secondary/association cortical regions (AudD/AudV, V2, S2 and M2) participate in later stages of hierarchical information processing, and likely integrate many inputs over a longer timeframe. We reasoned that these differences in processing needs may be reflected in a differential balance of perisomatic interneurons. To see if the relative content of perisomatic interneurons differs between primary and secondary areas of the cortex, we calculated the subtractive difference between the average CCK-GABA cell percentage and the average PV-GABA cell percentage (termed *D* score) in all brain regions studied (**Figure 7**). Notably, this analysis is distinct from those conducted previously, as it directly compares CCK- and PV-GABA cells to each other and does not include other types of GABA cells.

Graphing the *D* score by rank revealed several interesting trends of CCK-GABA cell distribution. The *D* score of any secondary/association cortical area (*green bars*) was always higher than the *D* score of the corresponding primary cortical area (*red bars*). This trend was observed in the case of the occipital cortex (V2mm/V2ml/V2L versus V1b/V1m), auditory cortex (AudD/AudV versus Aud1), sensory cortex (PtaL/PtaM/S2 versus S1bf/S1tr/S1ulp) and motor cortex (M2 versus M1). This finding suggests that CCK-GABA cells tend to comprise a greater proportion of the perisomatic interneuron population in secondary/association cortex than in the primary cortex. In contrast, PV-GABA cells tend to comprise a greater proportion of the GABA cells in the primary cortex, a result consistent with prior antibody labeling studies (Cruikshank et al., 2001).

## Discussion

To our knowledge, the current study represents the first comprehensive, systematic analysis of CCK- and PV-GABA interneuron distribution in the forebrain. Using a dual recombinase-based genetic strategy, we quantified two major populations of perisomatic interneurons in a wide volume of discrete brain regions. This extensive sampling contrasts with the focus of previous studies, which examined fewer areas (Hendry et al., 1984; Celio, 1986; Demeulemeester et al., 1988; del Rio et al., 1994; Kawaguchi and Kondo, 2002; Jinno and Kosaka, 2003a,b; Tamamaki et al., 2003). By broadly quantifying these cells, we identified novel trends in their distribution. CCK-GABA cells were shown to be remarkably abundant in several brain regions outside of the hippocampus, including the medial prefrontal cortex and ventrolateral temporal cortex. Additionally, CCK-GABA cells tended to be more abundant in secondary/association areas of the cortex than in primary sensory areas.

Overall, the distribution of CCK- and PV-GABA cells detected in the current study was consistent with that demonstrated by antibody labeling studies. While the percentage of PV-GABA cells was in agreement with past reports, the percentage of CCK-GABA cells in our study was higher. There are two explanations for this discrepancy. The first possibility is that previous studies may underestimate the number of CCK-GABA neurons due to a low efficacy of antibody labeling for CCK, as has been suggested by Gallopin et al. (2006) and implied by the results of Xu et al. (2010). In contrast to antibody labeling, the expression of GFP reporter in the CCK-*Frepe* mouse is highly sensitive to CCK transcript expression and readily occurs even in CCK-GABA neurons containing a very small amount of CCK peptide. An alternative possibility as to why CCK-GABA cells are more numerous in our model is that GFP-labeling may not be entirely exclusive to CCK-GABA cells. Though GFP-labeling reflects the expression of the CCK transcript, some cells which express the CCK transcript only do so temporarily and do not actually produce the CCK peptide (Tricoire et al., 2011). Therefore, a portion of GFP-labeled cells in this study may represent nonCCK-GABA cells that have a history of transient CCK expression. However, our specificity data suggests these nonCCK cells may only represent a modest portion of GFP-labeled cells (∼15%). Even when excluding these cells from the CCK-GABA population, the adjusted percentage of CCK-GABA cells in many areas is still much higher than that suggested by prior antibody labeling studies. Accordingly, our data suggest that the population of CCK-GABA cells has been at least slightly underestimated by the literature.

The significance of the small interneuron populations (10–20% of all GABA cells) identified by the present study should not be underestimated. Importantly, several studies report that even modest populations of perisomatic interneurons may serve important functions. The CCK-GABA cells of the basolateral amygdala, which likely constitute <10–15% of the total GABAergic cells in the region (unpublished observation), play a vital role in regulating fear and its extinction (Bowers and Ressler, 2015). Other factors besides cell population size may be determinants of the functional relevance of an inhibitory network, such as the volume of post-synaptic connections exhibited by each cell. A single CCK- or PV-GABA cell, for example, may form synapses on thousands of pyramidal cells. Given the ability of individual perisomatic interneurons to influence a large proportion of the pyramidal cell network (Cobb et al., 1995), even small populations of these neurons may shape information processing and therefore behavior.

The functional significance of the relatively large populations of CCK-GABA cells within the medial prefrontal and ventrolateral temporal cortices has not been determined. An attractive possibility is that these neuronal populations may help fine-tune working memory processes. CCK-GABA neurons commonly express CB1 receptors, which have been consistently implicated in working memory performance in humans and animals (Varvel and Lichtman, 2002; Han et al., 2012; Ruiz-Contreras et al., 2014). Theoretical modeling suggests that CCK-GABA neurons are ideally suited to regulate working memory because they exhibit depolarization-induced suppression of inhibition (DSI), a form of plasticity mediated by CB1 receptors (Wilson and Nicoll, 2001; Wilson et al., 2001). In CCK-GABA neurons, DSI limits the release of GABA from the presynaptic terminal (Wilson and Nicoll, 2001; Wilson et al., 2001). Thus, DSI allows for CCK-GABA neurons to dynamically adjust the strength of inhibitory transmission to their post-synaptic targets, which include pyramidal cells involved in working memory processing. Therefore, DSI may allow pyramidal cells to maintain their activity – and presumably the working memory representations that are encoded by their activity – for prolonged time periods (Carter and Wang, 2007).

Interestingly, our comparative analysis suggested that the primary and secondary areas of the cortex display a differential balance of PV- and CCK-GABA cells. In secondary cortical regions, the value of CCK-GABA cell percentage minus PV-GABA cell percentage was consistently higher than in primary cortical regions. Currently, the significance of this relatively greater content of CCK-GABA cells to network activity is unclear. CCK-GABA cells may be particularly influential to information processing in these regions, as these cells may integrate signals over a prolonged timeframe (Bartos and Elgueta, 2012) and maintain local activity patterns using a DSI-dependent mechanism (Carter and Wang, 2007). This property is in keeping with the nature of the secondary cortex, which integrates a wealth of complex inputs from several sources. In contrast, CCK-GABA cells may be less effective for information processing in primary cortex regions, which generally participate in the early stages of sensory processing and must integrate and transmit signals quickly. In this regard, fast-spiking PV-GABA cells may be more advantageous (Jonas et al., 2004; Doischer et al., 2008). While the relative abundance of CCK-GABA cells in human cortex needs to be examined, their relative enrichment within the secondary cortices of mice likely has important implications for human populations, as the relative size of the secondary cortical areas is much greater in humans than in rodents.

The balance between CCK- and PV-GABA cells also has important implications for network processing, as CCK-GABA cells may synapse with PV-GABA cells in areas such as the hippocampus (Hu et al., 2010; Lee and Soltesz, 2011). In the hippocampus, CCK-GABA cells release the peptide transmitter CCK on to adjacent PV-GABA cells, thereby exciting them (Lee and Soltesz, 2011). Thus, CCK-GABA neurons may act as a ‘switch’ to initiate PV-GABA neuron activity. A loss of CCK-GABA neurons, such as that seen in neuropsychiatric disorders, may therefore disrupt the function of PV-GABA neurons and the functions governed by these cells. Accordingly, a change in the balance between CCK- and PV-GABA neurons may present as a risk factor for behavioral impairment in disease. It remains to be shown how the relationship between these neurons is affected in pathology, as most studies examine a single neuronal population.

The current study reveals a staggering array of GABAergic cell populations that currently have no identified function. The behavioral relevance of many large cell populations of perisomatic interneurons, such as the PV-GABA cells of the retrosplenial cortex, has yet to be studied directly. In this regard, optogenetic approaches combined with the dual recombinase-based neuron strategy may be useful, as this method permits the selective activation of genetically distinct cells with a high degree of spatial resolution. Optogenetic techniques have been applied to study of specific GABA neuron populations which contain somatostatin, vasoactive intestinal peptide and PV (Lee et al., 2013; Pi et al., 2013; Courtin et al., 2014), and could be of use in studying the interneuron populations shown here. Besides optogenetics, selective activation of specific GABA neuron populations may also be possible using pharmacogenetic techniques such as ‘designer receptor exclusively activated by designer drug’ (DREADD) mouse model (Nguyen et al., 2014; Perova et al., 2015).

Our data support the validity of the dual recombinase-based approach as a tool for conducting quantitative analysis of neuronal subpopulations. Though only CCK- and PV-*Frepe* transgenic mouse lines were utilized in the present study, the dual recombinase-based approach could also permit the visualization of different cell types such as neuropeptide Y-, vasoactive intestinal peptide- or reelin-expressing GABA neurons. This approach would greatly facilitate the study of specific interneuron populations, many of which are still poorly characterized. Further, the dual recombinase-based genetic strategy can be applied to study the role of specific GABA neurons in a diverse range of neural pathologies. Indeed, many disorders are characterized by profound changes in GABAergic cells or their signaling, including schizophrenia, maternal immune activation, stress, depression and autism (Peng et al., 2013; Perez and Lodge, 2013; Aldrin-Kirk et al., 2014; Godavarthi et al., 2014; Kim et al., 2014a; Knoferle et al., 2014; Ma and McLaurin, 2014; Tong et al., 2014; Zamberletti et al., 2014). In these disorders, quantitative changes in specific GABA neuron populations in discrete brain regions can be explored.

## Author Contributions

PW and JK wrote the paper. JC managed the animal colony, prepared brain sections and conducted immunohistochemistry. PW and NF collected all cell count data; PW performed all analysis.

## Ethical Statement

All procedures were performed in accordance with the guidelines of the National Institutes of Health (NIH) and the Canadian Council on Animal Care (CCAC) with approval from the University of Toronto Animal Care Committee.

## Conflict of Interest Statement

The authors declare that the research was conducted in the absence of any commercial or financial relationships that could be construed as a potential conflict of interest.
